# The power of powerful others: health locus of control and vaccination behavior in rural Namibian pastoralists

**DOI:** 10.1186/s44263-026-00251-4

**Published:** 2026-03-01

**Authors:** Sean Prall, Brooke Scelza, Aparicio Lopes

**Affiliations:** 1 Department of Anthropology, University of California, Los Angeles, USA; 2 OnePencil Namibia, Opuwo, Namibia

**Keywords:** Vaccination, Health locus of control, Market integration, Pastoralists

## Abstract

**Background:**

Who we believe controls our health, whether it is ourselves, chance, or powerful others, shapes how we make healthcare decisions. The health locus of control (HLC) framework has been key to understanding self-efficacy in healthcare behaviors. Health promotion often emphasizes self-efficacy in decision-making. However, in more traditional, subsistence based societies with low levels of market integration (the shift from traditional subsistence towards reliance on market-based consumption) self-efficacy may not be sufficient to understand health decisions. This is particularly true with regard to vaccine beliefs and behaviors, where evidence for the role of HLC is mixed, and in populations where entities associated with vaccines may be viewed with suspicion.

**Methods:**

Using a novel ranking task, we examined the association between HLC and vaccine interest and perceptions in Namibian agro-pastoralists (*N* = 293) across a spectrum of market integration. Market integration was estimated via a principal components analysis. Sets of Bayesian multi-level models were used to assess HLC on vaccination questions via a monotonic function.

**Results:**

*External* HLC dominated: *powerful others* (e.g., ‘doctors’) and *God* domains were ranked highest, while *internal* HLC (‘self’) was consistently lowest. Greater market integration was associated with higher *internal* HLC and lower *powerful others* HLC. Individuals who ranked ‘doctor’ highly expressed more pro-vaccine sentiments, whereas those who ranked ‘self’ highly were more skeptical of vaccines. No HLC ranks predicted COVID-19 vaccination status.

**Conclusions:**

Variation in HLC across levels of market integration suggests that exposure to broader economic and cultural systems shifts beliefs about health control, with external sources of control playing a more dominant role in more rural areas. In contrast to studies of HLC in industrialized populations, *internal* HCL was negatively associated with interest and perceptions of vaccine safety. Public health efforts that emphasize individual decision-making may not resonate as well in such contexts, whereas carefully considering the role of *powerful others* may improve outreach efforts.

**Supplementary Information:**

The online version contains supplementary material available at 10.1186/s44263-026-00251-4.

## Background

Vaccines remain one of the most effective tools for controlling infectious disease and are responsible for saving millions of lives every year [1]. However, in recent years, vaccine hesitancy and negative perceptions of safety have eroded vaccine uptake. Before the COVID-19 pandemic, some public health experts were already reflecting on the possibility that negative perceptions about vaccines, not access to vaccines, were the primary barrier to vaccine uptake [2, 3]. The situation has only worsened since 2020, resulting in a worldwide decline in perceptions of childhood vaccine effectiveness, importance, and safety in dozens of countries [4]. These trends underscore the urgent need to examine not only vaccine uptake but also the underlying sentiments and psychological motivations that drive it if we are to build on the public health successes of the last 50 years.

A myriad of factors influences healthcare decisions like the choice to vaccinate, from physical access to healthcare facilities to psychological and cognitive factors that influence the propensity to seek care. The health locus of control (hereafter HLC following convention by original authors, see [5]) is a psychological construct that has been extensively studied in the health behavior literature and is believed to play an important role in healthcare seeking behaviors. Instruments to measure this construct have a long history of development and testing, and have been used widely across health domains over the last four decades [6–8]. The main HLC prediction is that individuals who believe they have higher individual control over their own health (*internal* locus of control) should be more motivated to engage in health behaviors that enhance their health and longevity, while those who believe that their own health is ultimately up to external factors like luck (*chance* locus of control) should be less motivated to pursue preventative healthcare [5, 9].

Numerous studies find significant associations between HLC measures and health outcomes. *Internal* HLC is positively associated with a better diet, exercise, regular tooth-brushing, perceptions of mental and physical health, and reduced rates of smoking, psychological distress, reliance on healthcare, and even myocardial infarction [10–17]. Causally, these associations may be explained via higher social capital, more investment in health behaviors, and generally better lifestyle choices by those with higher *internal* HLC [13]. Conversely, *chance* HLC is associated with lower health information seeking, reduced dental care, poor diet, reduced exercise, and increased alcohol consumption [16, 18, 19]. While some substantive critiques have been leveled at the ways HLC has been examined and interpreted, it remains a useful concept in the health behavior literature to disambiguate between spheres of influence on health behavior [16].

Additional domains, including the belief that one’s health is under the control of powerful individuals such as doctors (*powerful others* locus of control), or under the control of * God and spirits*, or other supernatural phenomena, have more recently been added to studies of HLC [20]. Like *chance*, these domains are *external* forms of HLC. However, domains referencing doctors or God have less clear-cut predictions and more inconsistent associations with health behaviors [16]. For example, the belief that doctors determine one’s health may increase the propensity to follow their advice, but if this belief leads people to think doctors will cure them, they may be less likely to take up preventative practices as a result [9]. A similar logic can be extended to the *God and spirits* HLC domain, whereby belief in divine control over health may undermine the self-efficacy of the individual [21, 22]. Alternately, beliefs in divine control may empower individuals to take control over their own health (the personal empowerment hypothesis). Some evidence suggests that the trade-off between personal and divine control is dependent on the degree of religiosity [21]. In support of the personal empowerment hypothesis, one study finds that a belief in divine control led to increased feelings of personal control, resulting in greater expectations for a long life [23]. These findings are generally in line with the literature suggesting religiosity is associated with benefits to health and longevity [24, 25]. More HLC research that is inclusive of these domains is needed to better understand these effects, particularly in places where external loci of control are likely to predominate.

Vaccination decisions have also been explored in the HLC literature. However, there is reason to suspect that this health behavior may operate fundamentally differently with respect to HLC than other preventative behaviors like exercise or diet. Healthy diets, physical exercise, and avoidance of smoking or drinking all constitute continual efforts to invest in health and longevity. Vaccine decisions are largely one-off, and do not have the continuous costs of these other behaviors. As such, *internal* HLC may be less important in vaccine decisions. Additionally, health providers are required for the delivery of vaccines, unlike diet and exercise that can be undertaken on one’s own, suggesting that *powerful others* HLC may be crucial. The directional impact of doctors is also dependent upon the level of trust in healthcare providers and the pharmaceutical industry to produce and deliver safe and effective vaccines. If individuals are vaccine hesitant, *internal* HLC may actually be associated with lower, not higher, vaccine uptake, as individuals leverage their sense of personal control to avoid something they view as potentially harmful and dangerous. For example, Aharon et al. examine HLC with respect to childhood vaccination compliance [26]. They show that *powerful others* HLC predicts childhood vaccination, but that *chance* and *internal* HLC are mediated through other variables including vaccine attitudes. Thus, HLC with respect to vaccination is likely to be shaped by medical mistrust, knowledge, and beliefs about vaccines, as well as broader socio-political attitudes.

The role of HLC on vaccination beliefs, hesitancy, intent, and status has been studied across several contexts, with decidedly mixed results. In the context of COVID-19 vaccine decisions, *internal* HLC is negatively associated with vaccine intentions in European university students, and vaccination hesitant and resistant groups in Ireland and the UK had higher *internal* HLC scores [27, 28]. Results from these studies also find that those who score high on *powerful others* HLC tend to be less averse to vaccines. However, another study showed that respondents who scored high on *internal* HLC were less vaccine hesitant, while those who scored high on *chance* HLC were more vaccine hesitant [29]. Other studies find no relationship with HLC and COVID-19 vaccine hesitancy or intention to vaccinate [30, 31]. Conversely, the role of *internal* HLC on COVID-19 prevention behaviors is mediated by fear of COVID-19, such that *internal* HLC predicted prevention behaviors when fear of COVID-19 was high, but not when fear was low [32]. *External* HLC also predicts agreement with public health guidelines during COVID-19 [33]. Aside from COVID-19, *powerful others* HLC is positively associated with influenza vaccination and intention among nurses across several studies [34–36]. *Powerful others* HLC also predicts Hepatitis B vaccination acceptance and intent [37, 38]. However, *internal* HLC has opposing effects in these studies, and still others find no relationship between HLC and influenza vaccination [39]. Finally, in a recent scoping review of HLC and vaccine hesitancy in parents, Magi et al. [40] find that most, but not all, studies show an inverse relationship between vaccine hesitancy and *internal* HLC, but a positive relationship between vaccine hesitancy and *chance* HLC, while *powerful others* HLC consistently predicts vaccine adherence. Taken together, these findings point to a complex and often inconsistent relationship between HLC dimensions and vaccine-related attitudes and behaviors, signaling the need for more nuanced and context-specific investigations into how the dimensions of HLC, and the factors that mediate them, affect vaccine outcomes.

The difficulties in applying HLC to understand vaccination behavior are even more complex in low- and middle-income countries (LMICs). Structural, historical, experiential, and cultural factors are all likely to impact how vaccines and vaccine programs are perceived as useful agents to alleviate disease burdens. Although structural factors remain a hurdle [41], people in LMICs may differ in the psychological motivations to seek out vaccines when they are available. Mistrust of medical systems, personnel, and institutions, particularly when they are believed to be foreign actors, is common in Africa [42]. Historical experiences with colonial powers place many in a position to be suspicious of medical care. For example, Central African communities that endured a brutal colonial medical campaign in the mid-twentieth century have lower vaccine uptake and trust in medicine decades later [43]. Suspicions that vaccines are designed and used by whites to harm, unethically experiment, kill, or render Africans infertile are widespread [44]. Fear and refusal of vaccines have severely impacted health efforts in Africa, from Polio eradication efforts to Ebolavirus outbreak mitigation [45–47]. Lower education rates and knowledge of biomedicine may further drive mistrust of vaccines. Underlying psychological factors motivating health behaviors may also be shaped by historical and cultural beliefs and experiences in unanticipated ways. Thus, HLC domains and their relationships with vaccine attitudes and decisions may not translate between high-income countries and LMICs.

To date, there is only limited work on HLC in LMICs, particularly from rural areas. However, Alami et al. [48] compare HLC domains between several industrialized countries with data they collected with Tsimane forager-horticulturalists in Bolivia. They find that Tsimane have lower *internal* HLC compared to data collected in Japan and the UK, but a higher *chance* and *powerful others* HLC. Further, they find that *powerful others* HLC positively and *chance* HLC were negatively associated with biomedical healthcare treatment, whereas *internal* HLC had no effect.

The Tsimane data suggest that one factor that may play an important role in mediating HLC is market integration. Market integration (hereafter MI) is broadly defined to reference the socio-economic shift away from traditional subsistence and toward a reliance on market-based products, but also includes participation in wage labor, ownership and consumption of various market goods, use of services, and changes in attitudes [49]. MI has many downstream effects, including reductions in kin density of social networks, alterations in sharing networks, impacts on diet and body composition, and disease ecology, among others [50–53]. Results from the Tsimane suggest that the focus on *internal* locus of control seen in studies conducted in market-based industrialized countries may not translate well to traditional subsistence-based societies with low levels of MI. The emphasis on *internal* HLC may instead be a product of a stable, low-mortality environment seen in industrialized countries [54]. Other studies looking at the role of collectivism suggest that the degree to which individuals favor *internal* vs *external* loci of control varies by degree of interdependence. For example, one recent study showed that participants from individualistic cultures (e.g., Germany, Hungary) were more self-reliant during the COVID-19 pandemic, compared to those in collectivistic cultures (e.g., China, India) who were more likely to rely on *powerful others* [55]. Small-scale, subsistence-based cultures are generally more collectivistic [56], and therefore more likely to rely on *external* loci of control. Which form of *external* loci is most dominant is likely to reflect a range of sociocultural factors. Broadening the populations where we study psychological constructs like HLC is imperative for improving our understanding of how generalizable these measures are, and their relevance to public health interventions around the world [57].

In this study, we apply a multifactorial approach to investigate how HLC applies to vaccine decisions in Namibian pastoralists. We sampled across the Kunene region of northwest Namibia, an area home to several ethnic groups reliant on small-scale agro-pastoralism. This region is a useful place to study HLC and healthcare decisions for several reasons. First, most Kunene residents outside of urban areas practice traditional subsistence strategies, and our previous work indicates a strong MI gradient is present. Himba pastoralists, who comprise a large portion of our sample, have long experienced marginalization by colonial governments [58]. In our previous work, we found high levels of medical mistrust in this group, which are attributed to differences in treatment as a result of cultural norms surrounding dress, hygiene, and level of education [59]. Additionally, we have found that healthcare decisions in these communities reflect perceptions of majority behavior [60] and the influence of prestigious figures [61], indicating that people rely on others for health information and may be likely to have greater *external* HLC.

We first explore the variance in HLC responses using a novel ranking task. We predict that the level of MI and education should be particularly important variables affecting HLC, such that respondents with higher levels of MI and formal education should favor *internal* HLC as they are exposed to outgroup norms and biomedical models of health [48]. We also predict that greater socio-economic status (SES) should correspond to greater *internal* HLC, as wealthier individuals may be more likely to bear the burden of travel and healthcare costs, allowing them greater self-efficacy over their ability to seek healthcare.

Second, we examine the role of HLC responses on a set of questions about vaccination. Drawing from previous literature, we predict that the *powerful others* HLC domain should be associated with more positive vaccination beliefs and decisions. This may be particularly important for Kunene pastoralists, where vaccines are viewed as the products of foreign entities [62]. Conversely, we predict that *internal* HLC will be mediated by medical mistrust, where *internal* HLC should predict vaccination attitudes only in individuals with trust in healthcare entities. In contrast, *chance* and *God* HLC should be negatively associated with vaccine attitudes, as individuals high in these domains may feel that health outcomes are outside of the purview of individual actions.

## Methods

### Study population and sampling

This study was conducted in the Kunene region of northern Namibia. The Kunene spans approximately 115,260 km^2^, but with only a single hospital in the regional capital of Opuwo and around 20 rural clinics. As a result, access to healthcare is limited and difficult for many Kunene residents who often struggle to get transportation to receive care [63]. Namibian healthcare is also burdened with staffing issues, a low doctor-to-patient ratio, resulting in long wait times and negatively impacting access to and perceptions of healthcare [64].

The region is home to several ethnic groups, including Himba and Herero. Most rural residents rely on small-scale agro-pastoralism, supplemented by store-bought maize and other staples. In town, paid labor is more common, with most households having at least one source of earned income. Even in the urban area, few residents have electricity or plumbing in their homes. Small schools are spread throughout the region; however, most adults in rural areas have little to no formal education. In rural communities, traditional ancestor worship is commonly practiced, while residents living in town are more likely to practice some form of Christianity. However, as a result of missionary outreach, many residents blend the two belief systems. Use of traditional medicines to treat common illnesses is widespread and often preferred to biomedical treatments [65, 66].

**Fig. 1:**
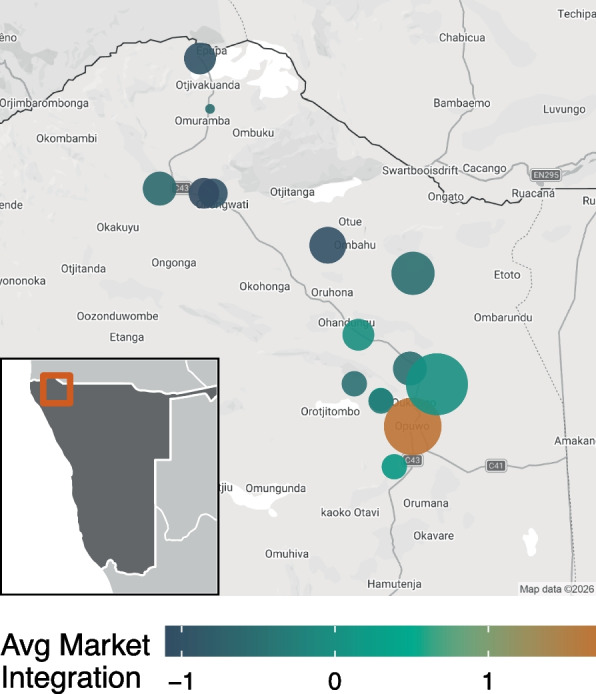
Sampling and participant residence map. Inset map indicates sampling area within Namibia. For each location, the average market integration estimate (standardized principal component) is shown in color, and sample size is indicated by point size. All locations outside of Opuwo are slightly jittered to protect the confidentiality of participants

We conducted a cross-sectional survey with participants across the rural–urban gradient, using a mix of randomized household selection and purposive sampling of communities. To collect a randomized sample, we generated a set of random GPS points across the study region. Next, we identified whether households were located nearby using satellite images from Google Earth, excluding locations unreachable by 4WD vehicle due to lack of roads or paths. If these randomly selected compounds were unreachable or unoccupied, we instead sampled from the next nearest compound. Opportunistic sampling was used for the urban sample, where participants were recruited from public markets and spaces in town. A map of participant locations across the study region is shown in Fig. 1. All data were collected during July and August 2024, and Otjiherero-speaking Kunene adults of any ethnic group were eligible to participate. All interviews were conducted in private in Otjiherero. Participants received small gifts of sugar, maize meal, washing powder, or cell phone minutes as compensation for their participation. This study follows relevant STROBE reporting guidelines, which are outlined in Supplementary material 1 [67].

### Demographics and market integration

All participants were asked for their age, tribal identity, residence location, and education. When required, age was assessed via the local year-name system [68]. Education was assessed by asking whether the participant attended any school, and if so, how many years of schooling they completed. Formal education among adults is still rare, with few participants in rural areas having completed more than a few years of formal education. Therefore, we use a binary variable denoting any formal education as our education measure for this analysis.

Wealth was measured using a modified version of the MacArthur ladder of subjective social status [69]. The original measure includes wealth, education, and job status as variables in the estimate, with the outcome on a 10-point ordinal scale. We use only wealth in our prompt, as previous piloting of this instrument indicated that Himba participants found the mix of variables confusing. This method was preferred over objective measures of wealth for several reasons. First, many Kunene pastoralists are reticent to list specific numbers of livestock they own. Wealthier individuals may not even know exactly how many animals they own, as they are often dispersed into remote cattle posts or are out on loan. Second, livestock wealth varies by level of MI, and people living in town are less likely to own livestock but more likely to have access to wage labor. Third, women typically do not own cattle but may have small numbers of goats and sheep. There may also be considerable variation in wealth between communities. As a result, to better compare perceived wealth across Kunene residents, a single subjective measure was used. Our modified question for the MacArthur ladder was “Compare yourself to other people living in your community. The wealthiest are at the top of the scale [10], the poorest are at the bottom [1]. Where would you place yourself?” While an imperfect measure of objective wealth, this measure best assesses how people judge their wealth relative to their peers of a similar lifestyle.

MI was assessed using a set of multifaceted questions about dwelling type (wall and roof materials), latrine type (bush/latrine/plumbed toilet), water source (sandwell, borehole, indoor plumbing), degree of traditional dress, and engagement in wage labor. Binary responses were then coded for each of these categories as either traditional or not. Although education may be viewed as an aspect of MI, we do not include formal schooling in this estimate, as we have predictions about education independent of MI. Variable responses were binary, so we used a convex logistic principal components analysis via the logisticPCA r package [70]. With this method we modeled a single principal component for the variables of interest, with higher estimates corresponding with a higher degree of MI. Additional details of this model are described in Supplementary material 1. Variable loadings are shown in Supplementary material 1: Fig. S1.

### Health locus of control ranking task

Most HLC research uses the multidimensional HLC scale, developed by Wallston et al. [71]. This instrument, which includes multiple HLC domains, uses more than 20 repetitive Likert scale items. Pilot testing in Himba communities indicated that participants were frustrated with the length and degree of repetition in this scale. Many of the items translated poorly, and participants found them difficult to answer, raising doubts about the ability to accurately capture HLC domains using this instrument. As a result, we devised a simple pile-sort task. In this task, participants were shown five cards with a graphical image depicting each domain: ‘chance,’ ‘doctor,’ ‘family,’ ‘God/spirits,’ and ‘self.’ These domains were chosen because they are similar to domains used in other HLC studies [48], and are culturally and conceptually relevant to this study. We consider ‘doctor’ to be the most similar to the *powerful others* domain used elsewhere. ‘Family’ was included as an additional *external* item, as kinship groups and kin networks are typically quite important in small scale societies like those sampled here. Each card was described to participants, followed by a comprehension check. Next, participants were asked to choose which card represented the domain they believed had the most control over their health. The first card selected was removed and received a rank of one. This process was repeated until all cards were ranked. It should be noted that this method significantly deviates from the multidimensional HLC scale typically used, and should be considered an adaptation of this measure, designed for cultural salience, not as a direct comparison of previous work.

### Health beliefs and behaviors

Participants were asked a series of questions about vaccine beliefs and attitudes. These included questions about existing and hypothetical vaccines, including COVID-19 vaccine status and interest in a potential malaria vaccine, using a mix of ordinal and binary response types. Question text is available in the Supplementary material 1. Finally, participants completed a shortened version of the group-based medical mistrust scale (GBMM) [72]. This instrument includes three subscales: suspicion, group disparities in healthcare, and lack of support from healthcare providers, and has been associated with vaccination decisions in industrialized settings [73]. To examine the association between medical mistrust and HLC on vaccine outcomes, we calculated average GBMM scores and used these to create a low medical mistrust and high medical mistrust group.

### Analysis

A set of Bayesian multilevel models were used to predict HLC rank and vaccination outcomes via the *brms* package in R using RStudio [74–76]. First, we wanted to understand which variables were associated with HLC rank, so HLC outcomes were jointly estimated using a multivariate cumulative ordered logit model. Second, to examine the associations between HLC rank and vaccine outcomes, all vaccine outcomes were jointly estimated using Bernoulli and cumulative ordered logit models using a multivariate framework. These models included varying intercepts for location, which were grouped into clusters of villages and compounds that fell within 10 km of each other, excluding the town of Opuwo. Predictors in these models include standardized age, engagement in formal education, a principal component estimate for MI, gender, subjective wealth (MacArthur Ladder score), and HLC ranks. As HLC ranks are not independent, individual HLC ranks were run in separate sets of models to compare the impact of individual HLC domains. Model comparison between each of these models and a null model without HLC predictors is calculated using PSIS-LOO [77]. Lastly, low and high medical mistrust groups were used as varying intercepts to predict vaccine outcomes, with varying slopes for ‘self’ HLC and ‘doctor’ HLC in independent models.

In all models, ordinal predictors, including HLC ranks, were assumed to influence outcome variables monotonically. These effects were estimated using the *mo()* function as part of the *brms* package [74]. For these monotonic effects, we plot the population-level posterior estimate that indicates the predicted difference between maximum and minimum categories (β_mo_, see [78]). Where relevant, we report the probability of distributions falling above or below zero (pr[β > 0] or pr[β < 0]). Additional packages used for data cleaning, location clustering, modeling, and data visualization include *tidyverse*, *janitor*, *dbscan*, *geosphere*, *tidybayes*, *modelr*, and *patchwork* [79–85]. Additional model details, posteriors, and other methodological information can be found in the supplemental material. Data and analytic code for the work described here are available at OSF [86].

## Results

**Fig. 2:**
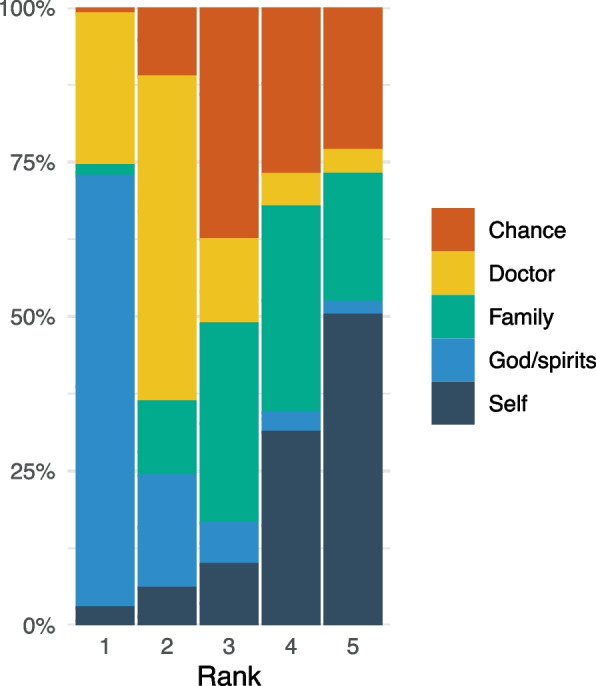
Distribution of HLC domains by rank

A total of 293 participants participated in the survey. Approximately 65% of the sample were Himba, while Herero and Zemba were equally the second most common at 7% of the sample. Eighteen percent of the sample took place in Opuwo, while the rest of the sample was collected between 10 and 167 km away. Average participant age was 37, and women comprised 60% of the sample. Sample characteristics are shown in Table 1. Nearly 70% of participants place ‘God/spirits’ in the highest rank, followed by ‘doctor’ at 25%. ‘Doctor’ was the most common second rank choice at 53%, while ‘chance’ was the most common third rank choice at 38%. Participants tended to rank ‘self’ last (50%). ‘Family’ was evenly distributed across ranks 3, 4, and 5, at 32%, 33%, and 21% respectively. Distribution of domains by rank is shown in Fig. 2.

**Table 1:** Sample characteristics

**Variable**	**Metric**	**Value**
**Age**	Mean	36.6
	SD	15.9
	Range	16-98
**Sex**	% Women	60%
**Any Education**	% With any education	34.5%
	Mean years of education	7.1
**Location**	% Town	18%
	% 0-50 km from Town	44.7%
	% 50-100 km from Town	16.3%
	% >100 km from Town	20%

### Predictors of HLC ranking task

**Fig. 3:**
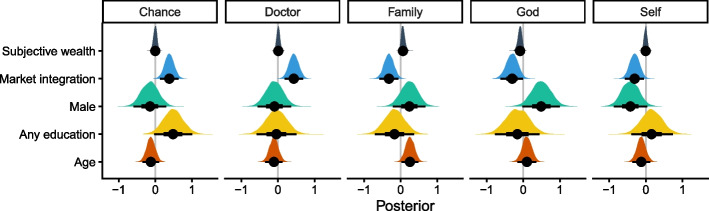
Posterior distributions of the predictors of HLC responses. Distributions that fall below zero indicate predictors are associated with higher ranks for a particular domain, while distributions that fall above zero are associated with lower ranks for a particular domain. The impact of subjective wealth was estimated using a monotonic function. Point and whisker plots show the posterior median, and 50% (thick line) and 95% (thin line) credible interval

A total of 278 participants completed the ranking task and answered all MI questions. Results from the multivariate cumulative ordered logit model indicated several meaningful predictors of HLC rank. The MI principal component influenced all HLC domains. More market integrated individuals tended to give ‘God,’ ‘self,’ and ‘family’ higher ranks (pr[$\upbeta$ < 0] = 97.3%, 99.1%, and 99.5% respectively), but give ‘doctor’ and ‘chance’ lower ranks (pr[$\upbeta$ > 0] > 99%, see Supplementary material 1: Fig. S2). Men tended to rank ‘self’ higher and ‘God’ lower than women (pr[$\upbeta$ < 0] = 96.7% and pr[$\upbeta$ > 0] = 97.3%). Participants with any formal education tended to rank ‘chance’ lower than those without schooling (pr[$\upbeta$ > 0] = 96.6%), but otherwise education had little impact on HLC ranks. Older respondents tended to rank ‘family’ lower than other domains (pr[$\upbeta$ > 0] = 98.4%). Subjective wealth was associated with higher rankings for ‘God’ (pr[${\upbeta }_{mo}$< 0] = 96%) but was not associated ‘self’ or any other domain. Posterior distributions of these predictors are shown in Fig. 3.

### Modeling perception of vaccines

Of the total sample, 253 participants answered all questions and listed a residence in a known location. Residences were grouped into 11 geographic clusters and included in the model as a varying effect. Responses to questions about vaccine perceptions and interest indicate the sample is generally positive about vaccinations. When asked if vaccines were generally safe, 95% of the sample agreed. Eighty-nine percent of participants felt that the COVID-19 vaccine was safe, although only 54% reported that they had received the vaccine. When asked to rate their interest in hypothetical vaccines for tuberculosis, malaria, and a novel, COVID-19-like disease, 89% reported being likely or very likely to receive the TB and malaria vaccines, and 91% reported that they would seek out the vaccine for a hypothetical disease.

**Fig. 4:**
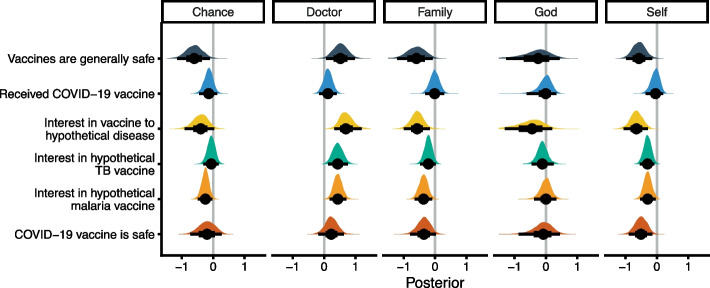
Posterior distributions of HLC on vaccine perceptions. Posteriors are reversed for ease of interpretation so that distributions that fall above zero indicate that higher HLC ranks (closer to 1) are associated with pro-vaccine outcomes, while distributions that fall below zero indicate that higher HLC ranks are associated with a lower probability of interest in vaccines or lower probability of affirmative responses to binary vaccine questions. Point and whisker plots represent the posterior median, and 50% (thick line) and 95% (thin line) credible interval

**Fig. 5:**
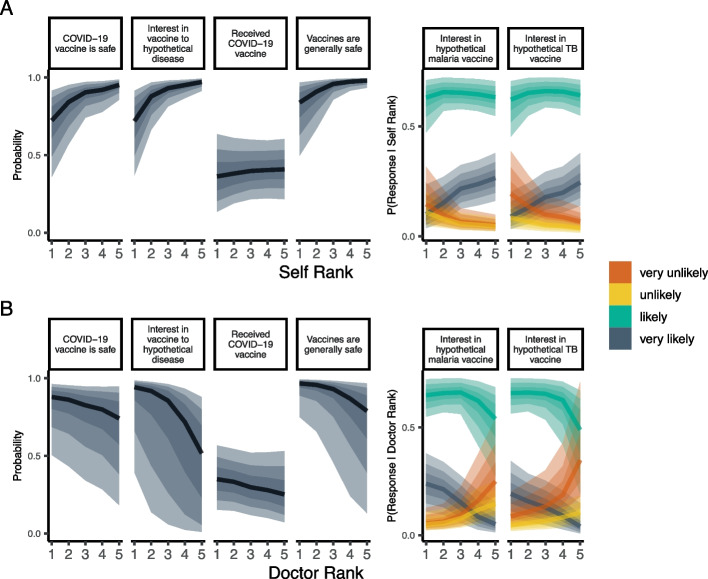
Posterior predictions for ‘doctor’ and ‘self’ item ranking on vaccine outcomes.** A** shows model predictions for ‘self’ rank on binary and ordinal outcomes, while **B** shows model predictions for ‘doctor’ rank. Color designates the Likert response for Likert items. Shading represents 50%, 80%, and 95% credible intervals

Jointly modeling the role of HLC rank on these vaccine outcomes indicates that several domains were predictive of vaccine beliefs and perceptions. Participants who rank ‘self’ higher were less likely to be interested in vaccines or believe that vaccines were safe (pr[${\upbeta }_{mo}$>0] > 99%), although there was no association between ‘self’ rank and whether they had received the COVID-19 vaccine (pr[${\upbeta }_{mo}$>0] = 62.9%). Conversely, participants who rank ‘doctor’ higher were more likely to be interested in vaccines, and believe that vaccines are generally safe (pr[${\upbeta }_{mo}$<0] > 98%), although ‘doctor’ rank little impact on receipt of the COVID-19 vaccine or views on the safety of the COVID-19 vaccine (pr[${\upbeta }_{mo}$< 0] = 79% and 87.4% respectively). ‘Chance’ and ‘family’ HLC ranks tended to be negatively, but inconsistently, associated with vaccine perceptions and interest. There was no consistent association between ‘God’ HLC rank and any vaccine outcome. Posterior distributions of the effect of HLC ranks on vaccine outcomes are shown in Fig. 4, and posterior predictions for the ranks of ‘doctor’ and ‘self’ shown in Fig. 5.

We then conducted a model comparison of the models including HLC ranks with a null model without HLC predictors. Model comparison indicates that the model containing ‘doctor’ HLC rank, followed by ‘self’ HLC rank, performed best. Models with other HLC ranks did not outperform the null model. Model comparison results are shown in Table 2.

**Table 2:** Model comparison results

Model	ELPD-LOO	ELPD difference	SE difference
Doctor	− 850.4	0.0	0.0
Self	− 866.3	− 15.9	8.7
Null model	− 869.7	− 19.3	9.5
Family	− 871.6	− 21.2	10.8
Chance	− 873.6	− 23.2	10.4
God	− 874.3	− 23.9	10.7

Models listed include noted HLC as a predictor, except for the null model which does not include any HLC predictor. ELPD-LOO is the expected log posterior predictive density of the LOO estimate. The ELPD difference column represents difference in the LOO estimate of the ELPD between the highest performing model and all other models. SE difference column shows the standard error of these differences

### Medical mistrust and HLC on vaccine outcomes

Multilevel models were used to compare the effect of high versus low GBMM scores on ‘self’ and ‘doctor’ HLC on vaccine beliefs and perceptions. Results of these models indicate few differences in HLC domains on vaccine outcomes between low and high medical mistrust groups. The largest effect on ‘doctor’ HLC rank can be seen in the difference between low and high medical mistrust groups for having received the COVID-19 vaccine, such that participants with low medical mistrust who rank ‘doctor’ highly were more likely to have received the vaccine than those in the high medical mistrust groups (% difference in posterior distribution = 92%). Other differences between these two groups are minor. Relevant posteriors for these models are shown in Supplementary material 1: Fig. S4 and posterior predictions are shown in Supplementary material 1: Fig. S5.

## Discussion

In this study, we assessed the relationship between our novel HLC ranking task and vaccine perceptions and decisions. Due to a dearth of data from small-scale populations, we began by looking at how the degree of *internal* vs *external* HLC compared to other populations. In sharp contrast to industrialized populations, our results show that Kunene pastoralists have very high *external* HLC, with the domain of ‘self’ receiving the lowest rank. This aligns with previous findings among Tsimane, another small-scale population, who also show more *externalized* HLC, especially when compared with industrialized samples from Japan and the UK [48]. Also similar to Tsimane, Himba ranked *powerful others* (‘doctors’) and the *God* domains highest. Together, these findings suggest that the dominance of *internal* HLC may be a common feature of industrialized populations but not representative of how small-scale societies feel about their health. However, as described below, we found that the ways that HLC mapped onto health behavior reflect both localized risks and limitations, as well as the particular cultural history of the region.

We found HLC to have important impacts on people’s perceptions of vaccines. Individuals who ranked ‘doctor’ (*powerful others*) as an important domain tended to have more pro-vaccine sentiments, while those who ranked ‘self’ highly were more skeptical of vaccines. Model comparison suggested that both the ‘doctor’ and ‘self’ rankings best predicted vaccine sentiment, while other domains did not outperform the null model. One interpretation is that individuals with a more *internalized* HLC are less susceptible to outreach and messaging by external institutions, internalize negative perceptions or experiences of these institutions, and are less susceptible to the imposition of health interventions by these institutions. If these institutions or interventions are viewed with suspicion, then people with greater *internal* HLC should leverage their sense of control over their own health to avoid them.

These results generally correspond with previously published literature. Studies on vaccine intention for HPV, COVID-19, influenza, and childhood vaccination also show that *powerful others* HLC tends to predict beliefs about vaccine safety and vaccine intention [28, 34, 35, 38, 40]. Similarly, other studies have shown that *internal* HLC was negatively associated with vaccination attitudes and interest, in line with our results [27, 40]. Overall, the vaccination questions showed very high trust and interest in vaccinations, a promising finding for future vaccine uptake in this community. Previous qualitative work with Himba and Herero pastoralists also suggested that, while they have a poor understanding of what vaccines are, childhood vaccination is normative and uncontroversial in these communities [62].

Despite its relationship with vaccine sentiment, we did not find any associations of HLC ranks on COVID-19 vaccine status. Additionally, none of our *external* HLC domains predicted perceptions of COVID-19 vaccine safety. However, a higher ranking of *internal* HLC (‘self’) was linked with more negative perceptions of COVID-19 vaccine safety. These results align with our previous work in the region, which showed that COVID-19 vaccination uptake was largely determined by access, and secondarily by fear or lack of interest in the vaccine [87]. Vaccines for COVID-19 are sometimes viewed with suspicion, and Kunene residents described weighing fear of the disease against fear of the vaccine. These results indicate that this skepticism may particularly affect healthcare decisions among those with higher *internal* HLC and provide further evidence that beliefs about COVID-19 and its vaccines do not always map well onto general perceptions of vaccine safety.

Another avenue we pursued in this paper was the effect of MI on HLC. Confirming our predictions, we found that greater MI was associated with higher *internal* HLC. This aligns with the findings by Alami et al. [48] that *internal* HLC tends to be more prominent in industrialized settings. Higher MI was associated with a lower rank of the ‘doctor’ (*powerful others*) domain. This may be the result of general distrust of doctors and medical personnel, particularly in urban and peri-urban contexts, negatively influencing the belief that they can influence health [59, 87]. We found suggestive evidence that implicates medical mistrust on the effect of the ‘doctor’ rank on COVID-19 vaccination status. Participants who ranked doctors as having a high degree of control over health were less likely to be vaccinated if they also had high medical mistrust, although differences were small.

The specifics of how MI affects HLC may be reflecting locally specific history and conditions. For example, our results contrast with Alami et al. [48], who found that Tsimane horticulturalists living closer to town had lower *internal* HLC and higher *chance* HLC, the opposite of our finding. This may be partially explained by formal education. Alami et al. [48] found that educational capital predicted greater internal HLC. We found no significant effects of formal education in our sample; however, the level of formal education is very low in our sample, with only 34.5% having attended any school. This may help to explain why education is the better predictor of *internal* HLC for Tsimane, while the more generalized MI measure we use is a better fit in the Kunene. Our results suggest a troubling trend whereby MI is correlated with a more *internalized* HLC, but an *internal* HLC is associated with skepticism of vaccines in these communities. As communities like those studied here will continue to become increasingly market integrated, additional work is needed to better understand this pattern.

While our findings largely comport with previous literature, there are several notable limitations to this study. Firstly, our HLC task is a novel ranking task, not the commonly used multidimensional HLC scale. Pilot testing indicated that this scale would be inappropriate for the Kunene, but our use of a novel ranking task limits comparability with other studies. Secondly, there are some conceptual and analytical issues with a ranking task, whereby participants were forced to rank some domains they might have otherwise placed on equal footing. Ranking also means that the individual domains are not independent, placing limitations on using these variables in an analysis. Despite these limitations, we feel that this approach better captures the locus of control concept in this population than would a long survey of Likert scale items. Additionally, contrary to our prediction, perceptions of medical mistrust only very weakly impacted the role of internal or powerful others’ HLC on vaccination outcomes. However, this may be a reflection of the particular medical mistrust scale used in this study. We use a subset of the group-based medical mistrust survey, which focuses on perceptions of medical care by race or ethnic group. This instrument was developed and validated in the USA in a sample of African American and Latina women, and is comprised of items that ask participants to think about the medical treatment of their ethnic group as a whole, not about individual experiences [72]. Previous work with Himba pastoralists suggests that they believe markers of cultural identity place them at risk of mistreatment in medical settings [59] and previous work in this area finds that other medical mistrust instruments focused on the individual predict vaccine perceptions [87]. Alternately, because this sample had generally high belief in vaccine safety, our ability to discern drivers of negative sentiments may be limited.

More specific to vaccination, the effects of HLC, measured using different methodologies, across different populations and on different vaccine outcomes is currently quite convoluted [26, 32]. There is a need to tease out the differences between vaccine hesitancy, intention, and decisions within and between HLC domains. As our data suggests, people think about different vaccines differently. Negative sentiment about COVID-19 may motivate internal control to refuse vaccination, while the reverse may be true for vaccines with less political baggage.

## Conclusions

Results from this and previous work applying the HLC concept to small-scale subsistence-based indigenous populations suggests that these populations think about the role of individual control very differently than do industrialized populations. They hold stronger beliefs in the role of powerful others like doctors, chance, or religious deities to control health. For example, in a previous study, we found that cultural beliefs about the etiology of malaria as an uncontrollable environmental force undermined the propensity to engage in preventative behaviors [66]. This raises issues for healthcare outreach and promotion, which tend to emphasize individual decision making. Including variables like medical mistrust can help us to understand how *powerful others* like doctors affect health behavior, particularly in populations where *external* domains of control are more prominent. Additionally, it is increasingly important to include measures of MI into studies of HLC in populations where people’s access to and beliefs in formal healthcare systems are likely to be rapidly changing.

Understanding drivers of healthcare behaviors, including psychological and cultural factors, remains crucial to increasing vaccine uptake and reducing the burden of infectious disease. This is particularly true in LMICs. Public health experts need to better understand how these populations think about their own ability to control disease. Outreach efforts that incorporate local models of health, in combination with further research understanding population differences, are likely to be both more culturally salient and more successful.

## Supplementary Information

Supplementary Material 1: Figure S1: Principal component model variable loadings. Figure S2: Posterior predictions for the role of market integration on HLC domains. Figure S3: Posterior distributions of all models predicting vaccine outcomes. Figure S4: Posterior predictions for models using self HLC Panel A shows model predictions for questions with binary outcomes. Panel B shows model predictions for questions using ordinal outcomes. Figure S5: Posterior predictions for models using doctor HLC Panel A shows model predictions for questions with binary outcomes. Panel B shows model predictions for questions using ordinal outcomes. Figure S6: Posterior distributions for varying intercepts and slopes of medical mistrust groups and HLC on vaccine outcomes Panel A shows model using self HLC, panel B shows model using doctor HLC. Results indicate no impact of applying HLC as a varying effect on low versus high medical mistrust groups. Figure S7: Posterior predictions for doctor HLC on low versus high medical mistrust groups on vaccine outcomes.

## Data Availability

A copy of the survey, as well as the data and analytic code associated with all work described in this manuscript are available on OSF via [https://osf.io/6cnbg/] (https://osf.io/6cnbg/?view_only=bd41db65ae39499bb634f8eca3242e98) or https://doi.org/10.17605/OSF.IO/6CNBG and are freely available [86].

## Abbreviations

COVID-19: Coronavirus disease of 2019
ELPD-LOO: Expected log posterior predictive density
GBMM: Group-based medical mistrust
HLC: Health locus of control
LMIC: Low- and middle-income country
MI: Market integration
SES: Socio-economic status
