# Supplementary material for: The power of powerful others: health locus of control and vaccination behavior in rural Namibian pastoralists
Source: BMC Glob Public Health. 2026 Mar 1;4:20. doi: 10.1186/s44263-026-00251-4 (PMC12950233; doi:10.1186/s44263-026-00251-4)
Supplement: Supplementary file 1 — Supplementary Material 1: Figure S1: Principal component model variable loadings. Figure S2: Posterior predictions for the role of market integration on HLC domains. Figure S3: Posterior distributions of all models predicting vaccine outcomes. Figure S4: Posterior predictions for models using self HLC Panel A shows model predictions for questions with binary outcomes. Panel B shows model predictions for questions using ordinal outcomes. Figure S5: Posterior predictions for models using doctor HLC Panel A shows model predictions for questions with binary outcomes. Panel B shows model predictions for questions using ordinal outcomes. Figure S6: Posterior distributions for varying intercepts and slopes of medical mistrust groups and HLC on vaccine outcomes Panel A shows model using self HLC, panel B shows model using doctor HLC. Results indicate no impact of applying HLC as a varying effect on low versus high medical mistrust groups. Figure S7: Posterior predictions for doctor HLC on low versus high medical mistrust groups on vaccine outcomes. [file 44263_2026_251_MOESM1_ESM.pdf]

Supplementary Material 1

The power of powerful others: Health locus of control and vaccination behavior in rural Namibian pastoralists

Sean Prall, Brooke Scelza, & Aparicio Lopes

Additonal methodological details

To assess vaccine beliefs, perceptions, and decisions, the following questions were used:

- Do you think that vaccines are generally are safe (y/n)
- Have you received the COVID-19 vaccine? (y/n)
- If not, why? (open ended)
- In your opinion, do you think that the COVID-19 vaccine is safe? (y/n)
- There is a new vaccine for malaria that is being developed. If that vaccine is available, how likely would you be to get it? (very unlikely/unlikely/likely/very likely)
- How likely would you be to have your kids get the malaria vaccine, if it becomes available? (very unlikely/unlikely/likely/very likely)
- There is a new vaccine for TB that is being developed. If that vaccine is available how likely would you be to get it? (very unlikely/unlikely/likely/very likely)
- Imagine there is a new illness similar to corona that is spreading around the world. Many people have gotten sick and died. The Namibian government is recommending getting vaccinated for this new disease. Now imagine that the doctors bring the vaccine to your compound. It is free to you and there is no line so you don't have to wait to get it. Would you get vaccinated? (y/n)

The group-based medical mistrust scale (GBMM), developed by Thompson et al. (2004) was used to measure medical mistrust. This scale includes 12 items with Likert-scale responses. In consultation with translators, we found that several of these items were difficult to differentiate when translated, and as a result very repetitive. Some items relied on foreign concepts and were not easily translatable (i.e "doctors and healthcare workers treat people of my ethnic group like 'guinea pigs' "). As a result, we only used 5 of the original 12 items in the GBMM (3 from the suspicion subdomain, and 1 each from the group disparities and lack of support domains) as listed below:

- 4. People of my ethnic group should be suspicious of information from doctors and healthcare workers.
- 5. People of my ethnic group cannot trust doctors and healthcare workers.
- 6. People of my ethnic group should be suspicious of modern medicine.
- 10. People of my ethnic group are treated the same as people of other groups by doctors and healthcare workers.
- 12. I have personally been treated poorly or unfairly by doctors or healthcare workers because of my ethnicity.

Principal component model for market integration

The following variables were compiled to use in the PCA:

- Participant dress
- Dwelling floor type
- Dwelling roof type
- Latrine type
- Water source

- Received cash for labor in the last year

For each variable, responses were coded into either traditional (i.e. traditional dress, cow dung and wood hut, with mud floor, no latrine, sandwell for water, no cash for labor) or modern (i.e. western dress, houses made from metal, brick, or concrete, latrine or plumbing, water from borehole or indoor plumbing, and has received cash for labor in the last year). These variables were then included in the PCA. A convex logistic principal components model explained 80.4% of the deviance in the data, and converged in 32 iterations. Loadings for variables are shown below.

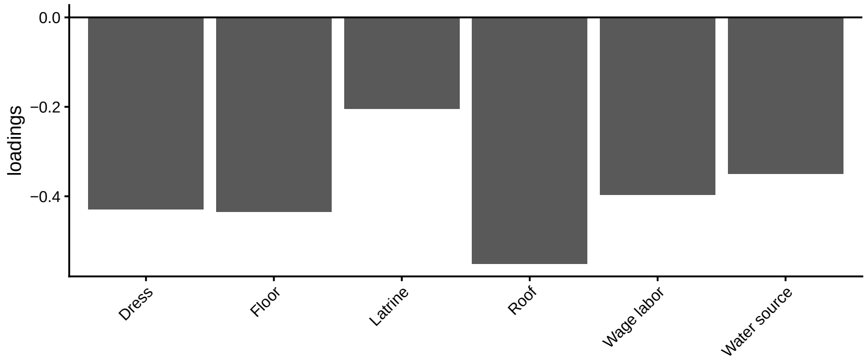

Figure S1: Principal component model variable loadings

Model to predict HLC

HLC ranked domains were jointly modeled using a cumulative ordered logit model, as defined below. The MacArthur ladder variable is ordinal, so monotonic effects were estimated using the *mo()* function in *brms*. These are represented below via d $\beta_l \cdot mo(ladder, \zeta_l)$. A full description of the use of monotonic effects applied to *brms* models can be found in Bürkner & Charpentier (2020).

$$\begin{matrix} self \\ doctor \\ god \\ chance \\ family \end{matrix} \sim OrderedLogit(\theta, \kappa)$$

$$\theta = \alpha + \beta_{Mi} \cdot MI + \beta_{age} \cdot Age + \beta_{male} \cdot Male + \beta_l \cdot mo(ladder, \zeta_l) + \beta_{edu} * Education$$

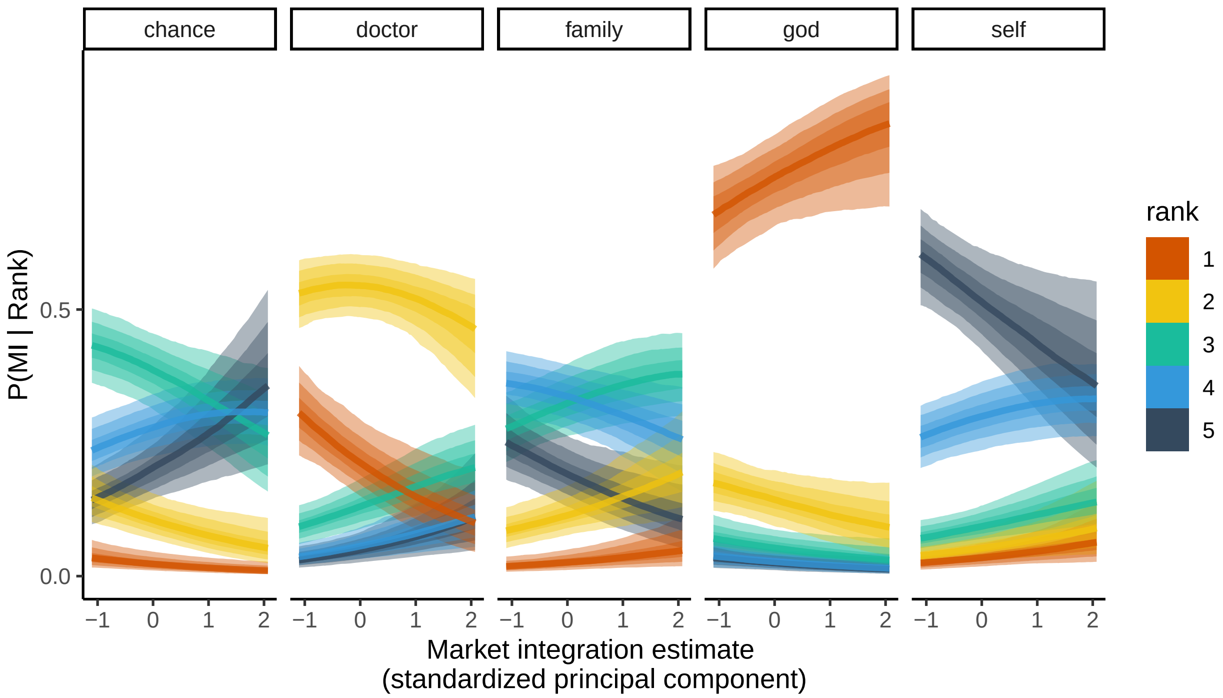

Figure S2: Posterior predictions for the role of market integration on HLC domains

Models used to predict vaccine attitudes

The following was used to jointly model vaccine interest and attitudes simultaneously for each HLC domain. Malaria and vaccine interest variables had ordinal responses, so a cumulative ordered logit model was used. The rest of responses were modeled using a bernoulli distribution. As the HLC domain and MacArthur ladder variables were coded as ordinal, monotonic effects were estimated using the *mo()* function in *brms*. These are represented below via $\beta_d \cdot mo(domain, \zeta_d)$ and $\beta_l \cdot mo(ladder, \zeta_l)$ respectively. Since HLC domains are linked, each domain predictor was run independently using this model.

$$\begin{matrix} Malaria\ vaccine\ interest \\ TB\ vaccine\ interest \end{matrix} \sim OrderedLogit(\theta, \kappa)$$

$$\begin{matrix} Vaccines\ safe \\ Hypothetical\ vaccine \\ COVID-19\ vaccine\ safe \\ Received\ COVID-19\ vaccine \end{matrix} \sim Bernoulli(p)$$

$$\begin{matrix} \theta \\ logit(p) \end{matrix} \sim \alpha + \beta_{male} \cdot Male + \beta_{age} \cdot Age + \beta_{edu} \cdot Edu + \beta_{Mi} \cdot MI + \alpha_{location} + \\ \beta_d \cdot mo(domain, \zeta_d) + \beta_l \cdot mo(ladder, \zeta_l)$$

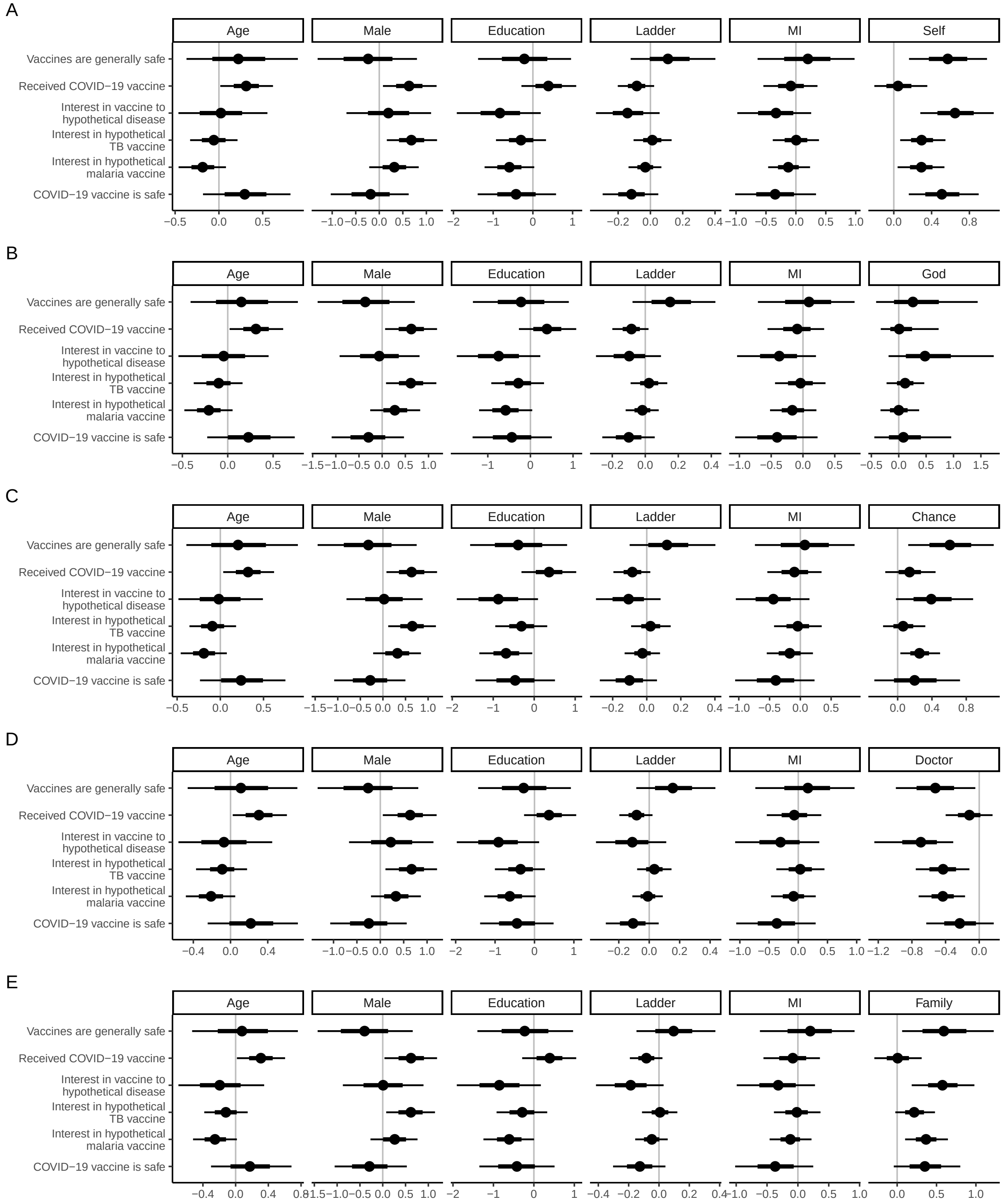

Figure S3: Posterior distributions all models predicting vaccine outcomes

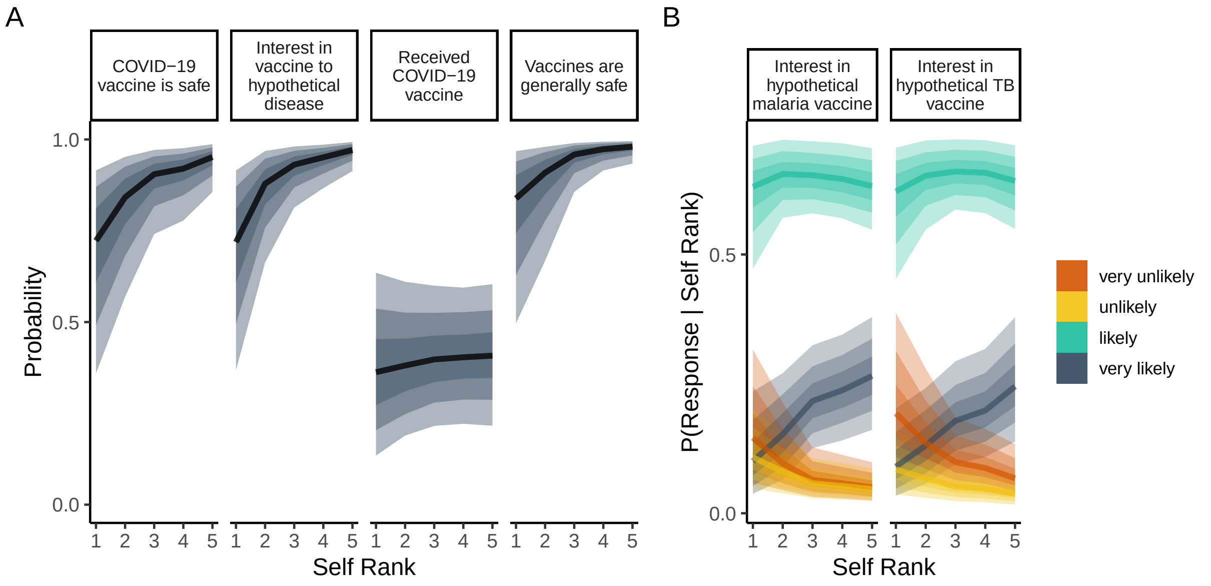

Figure S4: Posterior predictions for models using self HLC Panel A shows model predictions for questions with binary outcomes. Panel B shows model predictions for questions using ordinal outcomes.

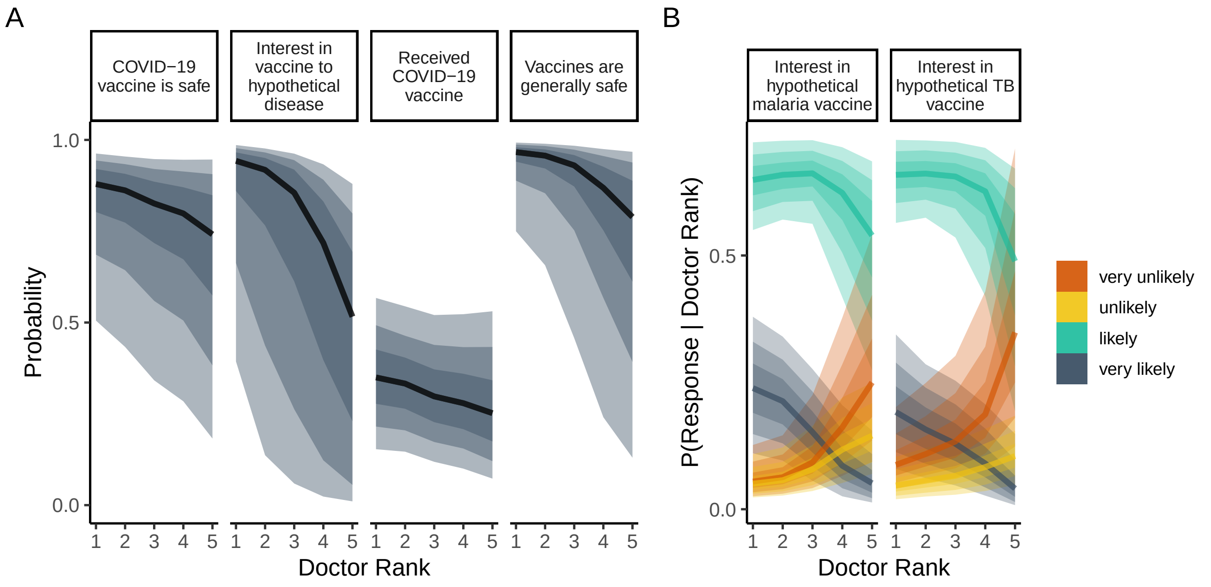

Figure S5: Posterior predictions for models using doctor HLC Panel A shows model predictions for questions with binary outcomes. Panel B shows model predictions for questions using ordinal outcomes.

Models used to predict whether HLC is mediated by level of medical mistrust

To examine the relationship of medical mistrust on the role of self and doctor HLC on vaccine attitudes, an average group-based medical mistrust score was calculated. Based on these averages, we created a low medical mistrust and high medical mistrust group via a mean split. These two groups were then incorporated into the previous model as a varying intercept, with HLC rank acting as varying slopes, again using a group-level and population level monotonic effect.

$$\begin{aligned} \overset{\theta}{logit(p)} &\sim \alpha + \beta_{male} \cdot Male + \beta_{age} \cdot Age + \beta_{edu} \cdot Edu + \beta_{Mi} \cdot MI + \alpha_{location} \\ &+ \beta_l \cdot mo(ladder, \zeta_l) + \alpha_{mm\ group} + (\beta_d + \beta_{d[mm\ group]}) \cdot mo(domain, \zeta_d) \end{aligned}$$

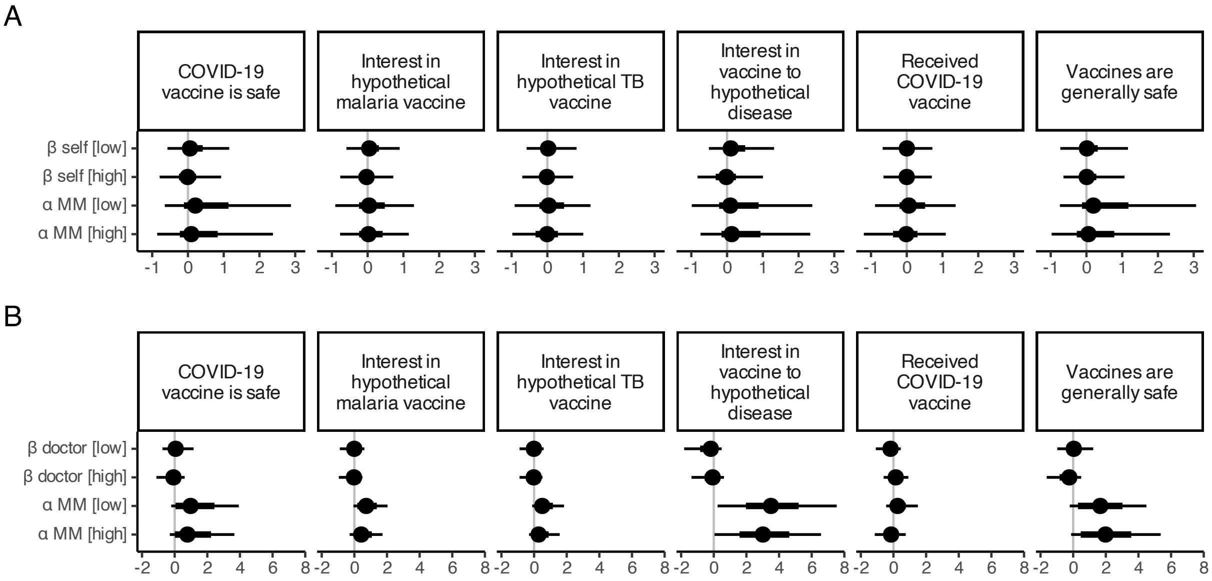

Figure S6: Posterior distributions for varying intercepts and slopes of medical mistrust groups and HLC on vaccine outcomes Panel A shows model using self HLC, panel B shows model using doctor HLC. Results indicate no impact of applying HLC as a varying effect on low versus high medical mistrust groups.

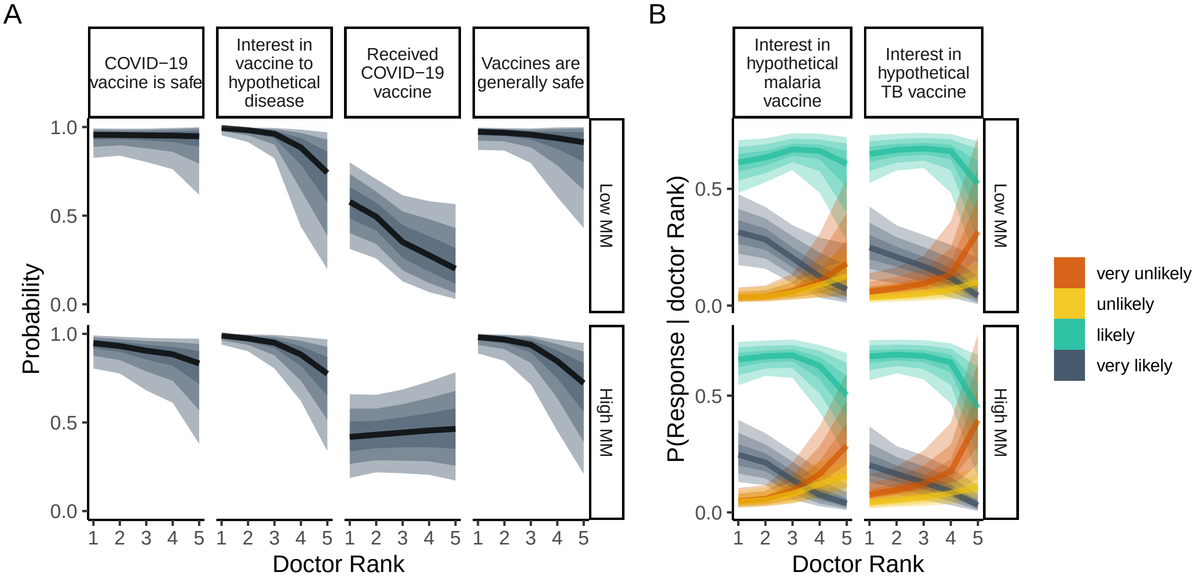

Figure S7: Posterior predictions for doctor HLC on low versus high medical mistrust groups on vaccine outcomes

Additional modeling details

All multilevel models were run in the *brms* package (Bürkner 2017). Model convergence was assessed by inspecting $\hat{r}$ values. All models used 4000 iterations, half of which were warm-up, and run on three chains. Regularizing priors were used for all intercepts ($\alpha \sim$ Normal[0,1]), predictors ($\beta \sim$ Normal[0,1]), and variance components ($\sigma \sim$ half-cauchy[0,1]). Default monotonic Dirchlet priors were used for all predictors with monotonic effects.

STROBE checklist (following von Elm et al. 2007)

Name	Item	Location	Notes
Title and abstract	1	pg 1-2	
Background/rationale	2	pg 3-9	
Objectives	3	pg 9-10	
Study design	4	pg 11	
Setting	5	pg 11-12	
Particiapnts	6	pg 11-12	
Variables	7	pg 12-15	
Data sources/measurement	8	pg 12-15	See also Supplementary Material 1
Bias	9	pg 11-12	
Study size	10		Opportunistic sampling
Quantitative variables	11	pg 15-16	
Statistical methods	12	pg 15-16	No subgroups examined. No sensivity analysis conducted.
Participants	13	pg 16-18	
Descriptive data	14	Table 1 and pgs 16-17	
Outcome data	15	pg 17-18	

Name	Item	Location	Notes
Main results	16	pg 16-20, Figures 2-5	
Other analyses	17		No subgroups
Key results	18	pg 20-21	
Limitations	19	pg 23-24	
Interpretations	20	pg 25	
Generalizibility	21	pg 23-24	
Funding	22	pg 27	

References

Bürkner, Paul-Christian. "Brms: An R Package for Bayesian Multilevel Models Using Stan." Journal of Statistical Software 80, no. 1 (2017): 1–28. https://doi.org/10.18637/jss.v080.i01.

Bürkner, P.-C. & Charpentier, E. Modelling monotonic effects of ordinal predictors in Bayesian regression models. British Journal of Mathematical and Statistical Psychology 73, 420–451 (2020).

Thompson, H. S., Valdimarsdottir, H. B., Winkel, G., Jandorf, L. & Redd, W. The Group-Based Medical Mistrust Scale: psychometric properties and association with breast cancer screening. Preventive Medicine 38, 209–218 (2004).

von Elm, E. et al. The Strengthening the Reporting of Observational Studies in Epidemiology (STROBE) statement: guidelines for reporting observational studies. The Lancet 370, 1453–1457 (2007).
